# Optimization of cellulase production under solid‐state fermentation by a new mutant strain of *Trichoderma reesei*

**DOI:** 10.1002/fsn3.852

**Published:** 2018-10-29

**Authors:** Nazanin Darabzadeh, Zohreh Hamidi‐Esfahani, Parisa Hejazi

**Affiliations:** ^1^ Department of Food Science and Industry Faculty of Agriculture Tarbiat Modares University Tehran Iran; ^2^ School of Chemical, Petroleum and Gas Engineering Iran University of Science and Technology Tehran Iran

**Keywords:** cellulase, mutation, optimization, screening, solid‐state fermentation, *Trichoderma reesei*

## Abstract

Nowadays, the use of agricultural by‐products, as the cheap substrate for the production of value‐added products, is of high interest for the researchers and practitioners. Cellulase is a relatively expensive and a very important industrial enzyme where in this study was produced form rice by‐products under solid‐state fermentation. A new mutant of *Trichoderma reesei* was used for cellulase production. The effective variables were initially screened by “Plackett and Burman design.” Afterward, the main variables including moisture content, P source, incubation temperature, and incubation time were optimized by “one factor at a time design.” Finally, the resulting variables including 74% for moisture content, 2 g/L for K_2_ HPO_4_, 30°C of incubation temperature, and 4 days of incubation time were reported as the ultimate optimal condition for cellulase production.

## 1 INTRODUCTION

Cellulase, as a complex enzyme containing endoglucanase, exoglucanase, and beta‐glucosidase, acts to hydrolyze cellulose in order to be converted into glucose units (Ma et al., 2013). These subunits of cellulase synergistically hydrolyze the cellulosic substrate (Lavudi, Harinder, & Laximi, 2013). Of industrial importance on its role as a critical enzyme, the biosynthesis of celluase by microorganisms has been recently paid attention (Liming & Xueliang, 2004; Singhania, Sukumaran, Patel, Larroche, & Pandey, 2010). The very high potential of cellulase enzymes in the hydrolysis of cellulose compounds makes them to be highly functional in various industries such as cotton processing, acetate processing, extraction of green tea components, improvement of food texture, seeds fermentation, brewing, soybeans hulls separating, quality improvement of animals feed, detergents producing, and animal feed additive processing, as well as significantly ethanol production increasing from the lignocellulosic biomass (Shweta, 2015). Cellulase can be produced by various microorganisms. Fungal cellulases are simpler than bacterial cellulases. Due to their high enzyme productivity and safe applicability in industries, *Aspergillus niger* and *Tichoderma reesei* are the most important fungi cellulase producers under solid‐state fermentation (SSF) (Abdullah, Greetham, Pensupa, Tucker, & Du, 2016; Chahal, 1985) compared to the less economic traditional method (Singhania, Sukumaran, & Pandey, 2007). It is mostly because of the cost of substrate consisting the major fraction of the total costs of cellulase production. Generally, in the submerged fermentation, cellulose with different values of purity is used for fermentation that leads to higher prices of final product (glucose). However, SSF emphasizes the use of cheaper substrate. So the application of the cheap agricultural substrates in this system may cause a major price reduction. SSF leads to a more concentrated enzyme preparation and then more suitable for various applications (Mekala, Singhania, Sukumaran, & Pandey, 2008). On the other hand, this technology is a popular technique used to generate enzymes for some economic and operational benefits. These advantages include low investment costs for equipment and operations, high volumetric productivity, higher final product concentrations, higher product stability, lower space requirements, and easier downstream operation (Behera & Ray, 2016; Lavudi et al., 2013). The application of this appropriate technology along with SSF operation control is promising to achieve a superlative method in the production of cellulase (Singhania et al., 2007).

In this study, the efficient conditions of cellulase production under SSF were investigated. A new mutant strain of *T. reesei* was applied for fermentation of rice by‐products. The components of medium and environmental variables influencing cellulase production were optimized in two stages using statistical design of experiments. Initially, screening was carried out among seven variables. Four parameters with the highest effect on cellulase production were identified. Later, the significant variables were optimized.

## 2 MATERIAL AND METHODS

### 2.1 Microorganism

A new mutant strain of *T. reesei* CECT 2414 was used in this study. In our previous study, *T*. *reesei* CECT 2414 was mutated by γ irradiation. In this manner, one of the mutants showed improved features such as more enzyme activity of its produced cellulase. This new mutant strain was called *T. reesei* B (Darabzadeh, Hamidi‐Esfahani, & Hejazi, 2018). A spore suspension was prepared by adding sterile saline solution on PDA medium containing the spores of *T. reesei* B. The final spore suspension was adjusted based on statistical design.

### 2.2 Substrate and SSF

In our previous study, different substrates from the by‐products of rice (rice bran, rice husk, and rice straw) were investigated. The results showed that a mixture of these three substrates (at the same ratio) may result in the best solution. It should be noted that the selected substrates were obtained from north of Iran were milled (Moulinex la picadpra, 700 W, Indonesia) and screened by sieving (mesh size: 14–18) (Darabzadeh et al., 2018).

For every treatment, 5 g of substrate was weighted and added to a 250‐ml flask. The proper water level was added to the compound. After sterilization at 121°C for 20 min, it was inoculated with the spore suspension. Next, it was incubated at a range of temperatures for a period of time, depended on statistical design (Darabzadeh et al., 2018; Li et al., 2010; Miller, 1959; Rocky‐Salimi & Hamidi‐Esfahani, 2010).

### 2.3 Enzyme extraction and assay

Cellulase was extracted by adding distilled water. After incubation, the weight of the fermented solid substrate was determined. Then, distilled water of about five times of this value was added to the flask. It was stirred for 30 min at 700 rpm (MR 3001 k Heidolph Germany). Subsequently, the suspended material and the biomass were separated by centrifugation at 10,000 × *g* for 15 min (3–30 K sigma Germany). The supernatant of this mixture was separated as cellulase solution. Enzyme activity was measured as filter paper activity (FPase) by Ghose method. In this regard, filter papers were used as the substrate. Cellulase solution was incubated at 50°C for 30 min. Afterward, the liberated reducing sugars in the reaction were determined by dinitrosalicylic acid reagent presented by Miller (Darabzadeh et al., 2018; Li et al., 2010; Miller, 1959; Rocky‐Salimi & Hamidi‐Esfahani, 2010).

### 2.4 Screening of parameters affecting cellulase production by “Plackett and Burman design”

The SSF production of cellulase is influenced by various factors, including media components and environmental parameters. The optimization of these parameters was performed in two steps. Initially, seven variables were screened by Modde 7 software. Then, the main parameters with a significant effect on cellulase production were selected. At the second step, these effective variables were optimized using one factor at a time design.

A “Plackett and Burman design” was used for screening by Modde 7 software. Incubation temperature, moisture content, mineral salts, inoculation concentration, incubation time, and concentration of K_2_HPO_4_ and nitrogen (ammonium sulfate, yeast extracts, and urea) in the medium were tested. These variables were screened at two levels in a design with seven parameters. The design matrix with eight experimental runs is shown in Table 1. The variables were investigated at two higher and lower levels designated as +1 and −1, respectively. The actual and coded values tested for each parameter are given in Table 2. The actual values of these levels were based on previous papers (Latifian, Hamidi‐Esfahani, & Barzegar, 2007; Mekala et al., 2008; Singhania et al., 2007). The experimental runs were performed, and the enzyme activities of cellulase were determined. The value of FPase was then recorded.

**Table 1:** “Plackett and Burman design” matrix for screening variables influencing cellulase production and Filter Paperase (FPase) of each treatment

Number	Mineral salts	Incubation time	P source	N source	Inoculation concentration	Moisture content	Incubation temperature	Filter paper activity (U/g dws)
1	+1	+1	−1	+1	−1	−1	+1	0
2	+1	−1	+1	−1	−1	+1	+1	1.8
3	−1	+1	−1	−1	+1	+1	+1	1.35
4	+1	−1	−1	+1	+1	+1	−1	1.13
5	−1	−1	+1	+1	+1	−1	+1	0.58
6	−1	+1	+1	+1	−1	+1	−1	1.41
7	+1	+1	+1	−1	+1	−1	−1	0.31
8	−1	−1	−1	−1	−1	−1	−1	0.15

*Note*

The variables were tested at two levels: the higher level designated as +1 and the lower level designated as −1.

**Table 2:** Actual levels of variables tested with Plackett and Burman and Effect estimate of variables on cellulase production

Parameter name	High level (+1)	Low level (−1)	Effect estimate
Temperature (°C)	35	20	0.1825
Moisture content (%)	75	55	1.1625
Inoculation concentration (spore/ml)	10^9^	10^6^	−0.0025
N Source (ammonium sulfate, yeast extract, and urea) (g/L)	1.7	0	−0.1225
P source (K_2_HPO_4_) (g/L)	2	0	0.6375
Incubation time (days)	6	3	−0.1475
Mineral salts	According to the method of Mandels and Weber^a^	0	−0.0625

a The solution is as follows: CaCl_2_ (0.3 g/L), MgSO_4_.7H_2_O (0.3 g/L), Tween‐80 (2 ml/L), and proteose peptone (1 g/L).

The mineral salts were selected according to the method of Mandels and Weber (1969). The solution (with a few changes) is as follows: (NH_4_)_2_SO_4_ (1.4 g/L), K_2_HPO_4_ (2 g/L), urea (0.3 g/L), CaCl_2_ (0.3 g/L), MgSO_4_.7H_2_O (0.3 g/L), Tween‐80 (2 ml/L), yeast extracts (0.25 g/L), and proteose peptone (1 g/L) (Mandels & Weber, 1969).

### 2.5 Optimization of significant parameters for cellulase production improvement

The significant parameters identified by the “Plackett and Burman design” were optimized using a one factor at a time method at five levels. The moisture content, K_2_HPO_4_ concentration, incubation temperature, and incubation time were the most effective variables representing significant influence on cellulase activity.

#### 2.5.1 Optimization of moisture content

For optimization of moisture content, a range of 65%–77% at five levels was used, while other conditions were fixed. These conditions are as follows: temperature of 35°C, incubation time of 3 days, presence of K_2_HPO_4_ in medium, inoculation concentration 10^7^ spore/ml, and absence of nitrogen and salts (Latifian et al., 2007).

#### 2.5.2 Optimization of K_2_HPO_4_

Optimization of K_2_HPO_4_ in the medium was carried out by adding it in a range of 1–5 g/L at five levels. The moisture content was adjusted to the optimized value from Section 2.5.1. The other conditions are similar to those in the previous section (Mandels & Weber, 1969).

#### 2.5.3 Optimization of incubation temperature

Incubation temperature was optimized in a range of 20–40°C. At this step of optimization, the values of moisture content and K_2_HPO_4_ were adjusted according to the optimized amounts of previous steps. The other conditions were similar to the previous efforts (Latifian et al., 2007; Mandels & Weber, 1969).

#### 2.5.4 Optimization of incubation time

The final stage of optimization process was devoted to finding the best incubation time. Therefore, moisture content, K_2_HPO_4_, and incubation temperature were adjusted according to the optimized amounts obtained from previous sections. Incubation time of 2–6 days was investigated, and the best of them was identified.

### 2.6 Statistical analysis

All experiments were carried out in triplicate, and all the cellulase activities were represented as the mean ± standard deviation of three identical values. To compare the data, analysis of variance (ANOVA) was performed using SPSS 18. The comparison between the mean values was done using the Duncan multiple range tests with a significance level of *p* lower than 0.05. Screening was done by “Plackett and Burman design” by Modde 7 software. The optimization of variables carried out by one factor at a time design.

## 3 RESULTS AND DISCUSSION

Environmental variables such as temperature, pH, water activity, nutrient, and O_2_ concentrations significantly influence microbial growth and product formation in SSF. So, determination of the appropriate level of these factors represents a high impact on the efficiency of enzyme production. Optimization of environmental variables and culture medium factors is a key concern for the development of suitable biotechnology for cellulase production (Mekala et al., 2008).

### 3.1 Screening of parameters affecting cellulase production

The aforementioned optimization process is noticeable in order for the productivity of cellulase production. For this purpose, Modde 7 software was used to validate the possible effects on enzyme activity by the aid of screening the variables. These primary variables included moisture content, incubation temperature, incubation time, N source, P source, mineral salts, and inoculation concentration (Table 1). In addition, the most effective factors were selected among the whole variables. Subsequently, four (out of seven) factors were selected due to the “Effect estimate” (Table 2).

### 3.2 Optimization of significant parameters for cellulase production improvement

The moisture content, P source, incubation temperature, and incubation time were the main variables with the greatest effect on cellulase activity. In the following, these parameters were optimized by one factor at a time design.

Each of these variables was evaluated for an estimated range from previous papers. However, other conditions in each treatment were set regarding to their negative or positive effects on cellulase activity (Table 2). The variables with negative effects on FPase were fixed on their low levels. Similarly, the parameters with a positive effect on cellulase activity set on their high levels (Table 2).

The moisture content had an effect estimate of +1.1625 (Table 2). The positive sign indicates that its high level (75%) leads to an increase in cellulase activity. The effect estimate of K_2_HPO_4_ was +0.6375 that showed it is less effective than water content; however, its positive effect indicates that cellulase activity was increased through the presence of K_2_HPO_4_. The third variable was incubation temperature with an effect estimate of +0.1825. The results showed that its effect on FPase is lower than two previous variables. However, incubation temperature has a positive effect on cellulase activity so its high level (35°C) increased the FPase. Incubation time was the last main variable. It has an effect estimate of −0.1475 expressing that its low level (3 days) has a significant effect on cellulase activity. It is also shown that cellulase activity was higher in 3 days compared to 6 days.

Cellulase production under SSF is affected by various variables including a combination of culture media and environmental parameters. Mekala et al. (2008) investigated the effects of incubation temperature, incubation time, and inducer concentration on cellulase activity. In this study, cellulase production was carried out by *T. reesei* RUT C30 with the sugarcane bagasse as a cheap substrate. At the first step, these three variables were selected by screening as the significant parameters on cellulase activity. Later, these variables were optimized by response surface method design (RSM) for obtaining the maximum cellulase production.

In 2017, Singhania et al. examined the effective variables on cellulase activity. In the first step, eleven factors were screened by the fractional factorial design. In the next step, the most effective variables were optimized by RSM. In this study, initial moisture content, particle size of substrate, pH of medium, incubation temperature, the age of inoculation, inoculation concentration, peptone concentration, ammonium nitrate concentration, inducer (cellubiose) concentration, Tween‐80 concentration, and incubation time and both low and high levels were investigated. The results of screening led to the selection of two variables including initial moisture content and incubation temperature. Last, the selected variables were optimized where *Aspergillus flavus* AT‐2 and *A. niger* AT‐3 were used. Incubation time, temperature, pH, moisture content, and C and N source were optimized.

#### 3.2.1 Optimization of moisture content

Since the moisture content had a significant increasing (positive) effect on FPase at its high level (75%), the range of moisture content was selected above 65% including 65%, 68%, 71%, 74%, and 77%. In this manner, the other variables were set, given their effects estimates. For example, P source was added in the medium because its effect estimate was positive. Other conditions were fixed as follows: incubation temperature of 35°C, incubation time of 3 days, inoculation concentration of 10^7^ spore/ml, and without N source.

Later, the FPase of these treatments was measured and the results are given in Table 3. Cellulase activity at 65% of moisture content was 0.206 U/g dws. It also levels up by increasing the amount of moisture content up to 0.881 U/g dws at 74%. This point was the best value for moisture content, reported as the optimum moisture content at 74%.

**Table 3:** Effect of moisture content on cellulase activity (U/g dws)

Moisture content (%)	Filter paper activity (U/g dws)
65	0.206 ± 0.06^c^
68	0.155 ± 0.05^c^
71	0.747 ± 0.01^b^
74	0.881 ± 0.02^a^
77	0.713 ± 0.07^b^

*Notes*

Statistically significant at 95% of confidence level.

Different letters indicate significant differences between the mean values within column.

The moisture content is different in a range from 30% to 85% in SSF, and this value has a significant effect on the growth of microorganism (Oriol, Raimbault, Roussos, & Viniegra‐Gonzales, 1988). The amount of moisture in the fermentation medium affects the microbial growth and biosynthesis. The moisture prepares the microorganism to take the substrate. Furthermore, efficiency of mass transfer in solid‐phase particles depends on the moisture and substrate characteristics; however, an excessive increase in moisture content inversely effects the enzyme production. It is because of the fact that more than its optimum amount of water leads to the reduction in the contact surface of the particles. As well, it thickens the water film resulting in less air supply to the particles. The solubility of the nutrients of the solid substrate, water absorption, and swelling of substrate are reduced due to the moisture contents of lower than its optimum value (Dutt & Kumar, 2014).

Lee, Darah, and Ibrahim (2011) studied the optimum level of moisture content for growth of *A. niger* USM AI 1 on sugarcane bagasse and palm kernel cake. Frequently, cellulase activity was reported in the moisture content of 70%.

Latifian et al. (2007) investigated the optimum moisture content for obtaining the maximum cellulase activity by using *T. reesei* QM9414 and *T. reesei* MCG77. The substrate was rice bran. In this study, moisture content, pH, and incubation temperature were optimized by RSM. Respectively, the optimum amount of moisture content for *T. reesei* QM9414 and *T. reesei* MCG77 were obtained 70% and 55%.

The optimization of cellulase production under SSF was explored by Singhania et al. (2007). Wheat bran was used as a cheap substrate. The moisture content and incubation temperature were optimized by central composite design. Regardless of incubation time, the maximum cellulase efficiency was measured at a moisture content of between 37% and 38%. The changes in moisture content did not affect the optimum temperature, indicating that there is no noticeable relationship between them. Because of the different substrate, the results in this study may not be compared by their work (Singhania et al., 2007).

Abdullah et al. (2016) used municipal solid waste for cellulase production under SSF by *A. niger* and *T. reesei*. Incubation temperature and moisture content were optimized. *T. reesei* significantly produced more cellulase compared to *A. niger*. The optimum moisture content was 60%. The moisture content affects the oxygen transfer and access to nutrients (Mrudula & Murugammal, 2011).

#### 3.2.2 Optimization of K_2_HPO_4_

Given the optimum moisture content of 74%, K_2_HPO_4_ was adjusted on 1, 2, 3, 4, and 5 g/L. Inoculation with a spore suspension (10^7^ spore/ml) was done after sterilization, and the samples were incubated in 35°C for 3 days. Finally, its FPase was measured (Table 4).

**Table 4:** Effect of K_2_HPO_4_ on cellulase activity (U/g dws)

K_2_HPO_4_ (g/L)	Filter paper activity (U/g dws)
1	0.82 ± 0.07^ab^
2	0.98 ± 0.10^a^
3	0.70 ± 0.10^b^
4	0.66 ± 0.18^b^
5	0.39 ± 0.04^c^

*Notes*

Statistically significant at 95% of confidence level.

Different letters indicate significant differences between the mean values within column.

Results showed FPase was 0.82 U/g dws in 1 g/L K_2_HPO_4_. Cellulase activity was increased to 0.98 U/g dws by increasing the K_2_HPO_4_ concentration to 2 g/L. However, as K_2_HPO_4_ concentration increased more than 2 g/L, the FPase decreased so the optimum K_2_HPO_4_ concentration was 2 g/L.

Abdullah et al. in 2016 added minerals to a solid substrate into a solution. In this solution, there existed K_2_HPO_4_ as a mineral source. Adding minerals did not affect the cellulase activity due to the existence of their sufficient amount in the solid substrate. In other words, minerals are not needed to be added.

#### 3.2.3 Optimization of incubation temperature

Incubation time was then evaluated once the optimum moisture content and K_2_HPO_4_ were given. Regarding the positive effect estimation of this variable and the existing literature on the range of desired temperature for fungi, five values of 20, 25, 30, 35, and 40°C were selected for optimization of temperature.

The substrate with the optimum moisture (74%) was prepared, and K_2_HPO_4_ concentration of 2 g/L was added to it. After inoculation, it was incubated for three days at different temperatures. So, FPase of these samples was measured (Table 5).

**Table 5:** Effect of incubation temperature on cellulase activity (U/g dws)

Incubation temperature (°C)	Filter paper activity (U/g dws)
20	0.00 ± 0.00^c^
25	0.06 ± 0.01^c^
30	1.16 ± 0.03^a^
35	0.85 ± 0.19^b^
40	0.00 ± 0.00^c^

*Notes*

Statistically significant at 95% of confidence level.

Different letters indicate significant differences between the mean values within column.

Incubation temperature had a significant effect on cellulase activity based on the results. At 20°C, no cellulase activity was achieved. Otherwise, by increasing temperature, the enzyme activity increased gradually and reached to its maximum (1.16 U/g dws) at 30°C. So this temperature indicated as optimum temperature. At more than 30°C, cellulase activity decreased, and finally, it became 0 at 40°C.

Temperature significantly affects the growth, development, and generally the metabolite's activity of an organism. At lower values than optimal temperature, the amount of cellulase activity decreased, which is probably due to the fact that at lower temperatures, there is no possibility of passing the substrate across the cell, which reduces the cellulase production efficiency. On the other hand, at higher temperatures, the energy needed to maintain cell growth is high due to denaturation of enzymes of the cell metabolism pathways reducing the production of metabolites (Dutt & Kumar, 2014).

Mekala et al. (2008) reported that incubation temperature was the second effective variable on cellulase activity. In their study, the optimum temperature of cellulase production by *T. reesei* RUT C30 was about 32–33°C Also their results showed different substrates affected the optimum incubation temperature. At optimum temperature and inducer concentration, the optimum incubation time was 66 hr.

Singhania et al. (2007) reported that *T. reesei* RUT C30 had the most cellulase production at 30–31°C. These results justify the results of present study.

Latifian et al. (2007) worked on optimizing incubation temperature, moisture content, and pH for cellulase production by *T. reesei* QM9414 and *T. reesei* MCG77 which FPase was 1.1635 and 2.314 U/g dws, respectively, at 30 and 25°C. These temperatures confirm the results of this study.

Abdullah et al. (2016) focused on optimizing cellualse production under SSF from municipal solid waste by *T. reesei* and *A. niger*. The results showed the best temperatures for *T. reesei* and *A. niger* were 30 and 25°C, respectively. Their results are consistent with this study.

#### 3.2.4 Optimization of incubation time

For the sake of optimizing incubation time, the substrate was prepared with 74% moisture and adding 2 g/L K_2_HPO_4_. After inoculation, the samples were incubated at 30°C for 2, 3, 4, 5, and 6 days. Then, FPase of these samples was measured (Table 6).

**Table 6:** Effect of incubation time on cellulase activity (U/g dws)

Incubation time (day)	Filter paper activity (U/g dws)
2	0.010 ± 0.009^c^
3	1.117 ± 0.034^b^
4	1.317 ± 0.018^a^
5	1.359 ± 0.032^a^
6	1.401 ± 0.042^a^

*Notes*

Statistically significant at 95% of confidence level.

Different letters indicate significant differences between the mean values within column.

Incubation time was effective on cellulase activity. After the second day of incubation, the FPase was 0.01 U/g dws. This value reached 1.117 U/g dws after 3 days of incubation. The most cellulase activity was in 4 days of incubation (1.317 U/g dws) meaning as the optimum time. By increasing incubation time to four and 5 days, we did observe no changes in cellulase activity. Therefore, in order to have a more economical and efficient process, the incubation time period of 4 days was chosen as the optimal time.

Dutt and Kumar (2014) produced cellulase using *A. niger* and *A. flavus*. The best time for these strains was 5 days. Cellulase activity after more than 5 days of incubation time decreased while biomass increased by up to 6 days. It is confirmed that enzyme production depends on biomass, but just at the exponential phase. Since cellulases are part of the primary metabolites, their production begins at exponential phase and decreases at death phase.

## 4 CONCLUSION

Cellulase production under SSF by a new mutant of *T. reesei* B using rice by‐products as a cheap substrate was studied in this paper. First, seven variables were screened by “Plackett and Burman design.” Accordingly, moisture content, K_2_HPO_4_ concentration, incubation temperature, and incubation time were determined as the most effective variables. In the next step, these variables were optimized by one factor at a time design. Based upon the results, the highest value in the optimized condition was assigned to the cellulase activity such that optimization variables were ultimately adjusted by 74% for moisture content, 2 g/L for K_2_HPO_4_ concentration, 30°C for incubation temperature, and 4 days for incubation time.

## CONFLICT OF INTEREST

The authors declare that they have no conflict of interest.

## ETHICAL STATEMENTS

This study does not involve any human or animal testing.
